# Automated Analysis of Spontaneous Facial Expression Variability in Neurocognitive Disorders During Naturalistic Conversation

**DOI:** 10.1002/alz70857_104472

**Published:** 2025-12-25

**Authors:** Peter S. Pressman, Mohammad Mahoor, Francesca R. Dino, Sofia S Messersmith, Erin S Marsh, Elliott D. Ross

**Affiliations:** ^1^ Layton Healthy Aging and Alzheimer's Disease Research Center, Portland, OR, USA; ^2^ University of Denver, Denver, CO, USA; ^3^ University of Colorado, Anschutz Medical Campus, Aurora, CO, USA; ^4^ University of Oklahoma, Oklahoma City, OK, USA; ^5^ University of Colorado School of Medicine, Aurora, CO, USA

## Abstract

**Background:**

A comprehensive investigation of facial expression variability during naturalistic conversations was conducted across individuals with behavioral variant frontotemporal dementia (bvFTD), Alzheimer's disease (AD), mild cognitive impairment (MCI), and healthy controls (HC). To avoid questions of accuracy of individual emotion identification, this study primarily employed automated facial expression analysis to quantify expression variability and diversity during social interactions.

**Methods:**

Participants engaged in 10‐minute recorded conversations about disagreements with family members, analyzed using a modified FER/MTCNN pipeline for facial expression detection. Emotional diversity was calculated using Simpson's Diversity Index, while dispersion measures captured variability in emotional intensities.

**Results:**

Results revealed that bvFTD demonstrated significantly lower emotional diversity (mean = 0.59, SD=0.03) compared to both AD (mean = 0.65, SD=0.03; d=‐2.15, *p* <0.001) and HC (mean = 0.57, SD=0.07; d=‐1.38, *p* = 0.006). BvFTD exhibited higher happy dispersion (mean = 0.92, SD=0.13) compared to HC (mean = 0.80, SD=0.18; d=0.80, *p* = 0.026), MCI (mean = 0.67, SD=0.23; d=1.69, *p* = 0.012), and AD (mean = 0.71, SD=0.19; d=1.47, *p* = 0.003). Categorical analysis revealed significant differences between diagnostic groups (χ^2^=17.52, df=9, *p* = 0.041), with AD showing predominant “sad” expressions (87.5%), bvFTD displaying varied patterns (58.3% sad, 33.3% neutral), and HC demonstrating the most balanced distribution (43.8% neutral, 37.5% sad, 18.8% happy). MCI uniquely showed no predominant sad expressions, instead exhibiting mostly neutral expressions (60%) and equal proportions of happy and angry expressions (20% each).

**Conclusion:**

These findings suggest that automated facial expression analysis can detect meaningful patterns of emotional variability across neurodegenerative conditions, potentially aiding in diagnostic differentiation. Future research should address current limitations through larger sample sizes, manual validation, and longitudinal designs to track changes over time.

